# Effects of Ramadan fasting on the diurnal variations of physical and cognitive performances at rest and after exercise in professional football players

**DOI:** 10.3389/fpsyg.2023.1148845

**Published:** 2023-03-28

**Authors:** Syrine Khemila, Mohamed Romdhani, Mohamed Amine Farjallah, Rihab Abid, Emna Bentouati, Mohamed Abdelkader Souissi, Salma Abedelmalek, Sergio Garbarino, Nizar Souissi

**Affiliations:** ^1^Research Unit Physical Activity, Sport and Health (UR18JS01), National Observatory of Sports, Tunis, Tunisia; ^2^High Institute of Sport and Physical Education, Ksar-Saïd, Manouba University, Manouba, Tunisia; ^3^Motricité-Interactions-Performance, MIP, UR4334, Le Mans Université, Le Mans, France; ^4^Université Sorbonne Paris Nord, Hypoxie et Poumon, H&P, INSERM, Bobigny, France; ^5^Département STAPS, Université Sorbonne Paris Nord, Bobigny, France; ^6^High Institute of Sport and Physical Education of Gafsa, University of Gafsa, Gafsa, Tunisia; ^7^Department of Neuroscience, Rehabilitation, Ophthalmology, Genetics and Maternal-Child Sciences, University of Genoa, Genoa, Italy; ^8^Post-Graduate School of Occupational Medicine, Università Cattolica del Sacro Cuore, Rome, Italy

**Keywords:** fasting, diurnal variation, fatigue, sleep, physical and cognitive performance

## Abstract

**Introduction:**

Ramadan fasting (RF) is characterized by daily abstinence from food and fluid intake from dawn to sunset. The understanding of the Ramadan effects on the diurnal variations of athletic and cognitive performance is crucial for practitioners, coach and researchers to prepare sport events and optimize performance. The aim of the present study was to reveal the effects of RF on the diurnal variation of physical and cognitive performances at rest and after exercise.

**Method:**

In a randomized order, 11 male football players (age: 19.27 ± 0.9; height: 1.79 ± 0.04 cm; body mass: 70.49 ± 3.97 kg; BMI: 21.81 ± 1.59 kg/m^2^) completed a 30-s Wingate test [i.e., mean (MP) and peak powers (PP)] at 07:00, 17:00, and 21:00 h on five occasions: 1 week before Ramadan (BR); the second (R2); the third (R3); the fourth (R4) week of Ramadan; and 2 weeks after Ramadan (AR), with an in-between recovery period of ≥72 h. Simple (SRT) and choice (CRT) reaction times, mental rotation test (MRT) and selective attention (SA) test were measured before and after Wingate test. Rating of perceived exertion (RPE), body composition, dietary intake, profile of mood states (POMS) and Pittsburgh Sleep Quality Index (PSQI) were assessed over the five periods.

**Results:**

Compared to BR, RF decreased MP at 17:00 h (*p* < 0.05, d = 1.18; *p* < 0.001, d = 2.21, respectively) and PP at 17:00 h (*p* < 0.05, d = 1.14; *p* < 0.001, d = 1.77, respectively) and 21:00 h (*p* < 0.01, d = 1.30; *p* < 0.001, d = 2.05, respectively) at R3 and R4. SRT (*p* < 0.001,d = 1.15; d = 1.32, respectively), number of correct answers (MRTE; *p* < 0.05, d = 1.27; d = 1.38, respectively) and SA (*p* < 0.01, d = 1.32; d = 1.64, respectively) increased during R2 and R3 in the evening before exercise compared to BR. Short term maximal exercise enhanced SRT (*p* < 0.01, d = 1.15; *p* < 0.001, d = 1.35, respectively), MRTE (*p* < 0.001, d = 2.01; d = 2.75 respectively) and SA (*p* < 0.05, d = 0.68; d = 1.18, respectively) during R2 and R3 in the evening. In comparison to BR, sleep latency and sleep duration increased during R3 (*p* < 0.001, d = 1.29; d = 1.74, respectively) and R4 (*p* < 0.001, d = 1.78; d = 2.19, respectively) and sleep quality increased in R2, R3 and R4 (*p* < 0.01, d = 1.60; *p* < 0.001, d = 1.93; d = 2.03, respectively).

**Conclusion:**

During RF, anaerobic and cognitive performances were unaffected in the morning but were impaired in the afternoon and evening. Short-term maximal exercise mitigates the negative effects of fasting on cognitive performance. Maximal exercise could thus partially counteract the effect of fasting on cognitive function.

## Introduction

Ramadan fasting (RF) is a fundamental rule of Islam during which healthy Muslim adults refrain from eating, drinking, smoking, chewing, and having sexual relations from dawn to sunset (Boukhris et al., 2019b). As a result, they generally consumed only two main meals (suhur, i.e., the last meal before starting the day’s fast, and, iftar, i.e., the meal taken to break the day’s fast). The requirement to eat only within the overnight span leads to several changes in meal composition and hydration state (Boukhris et al., 2019b). The changes associated with the acute diurnal dehydration that characterize RF may produce many effects upon athletes’ physiology and psychology which could have a negative impact on sport performance (Chtourou et al., 2011). Similar modifications in various hematological and biochemical parameters suggesting changes in hydration status and impairment in renal function were found during Ramadan, according to Trabelsi et al. (2018). Furthermore, in a recent meta-analysis, it has been shown that Muslim athletes report increased subjective feelings of fatigue, illness, lethargy, and mood changes during RF, which could lead to their inability to maintain physical effort, particularly during high-intensity exercises (Hsouna et al., 2020; Trabelsi et al., 2022a). In addition, the Ramadan-induced nocturnal food consumption led to reduced sleep quality and quantity in athletes (Faris et al., 2020; Romdhani et al., 2022c). In a recent systematic review and meta-analysis of studies involving athletes and physically active individuals, Trabelsi et al. (2022b) reported that athletes who continued to train during RF experienced a decrease in sleep duration, impairment of sleep quality, and increase in daytime nap duration. In another recent systematic review and meta-analysis, Trabelsi et al. (2020) reported that when athletes (aged ≥ 18 years) continued to train at least twice/week while observing RF, sleep duration was decreased compared with their baseline levels. Several studies found that acute sleep restriction disturb next-day physical and cognitive performances (Romdhani et al., 2019; Khemila et al., 2021).

Some studies have shown that strength and high-intensity aerobic and anaerobic performances and the long-duration incremental and non-incremental exercises are negatively affected (Chtourou et al., 2019; Correia et al., 2020), while others failed to identify a significant decrement of physical performance during RF (Zarrouk et al., 2016; Boukhris et al., 2019b; Abaidia et al., 2020). The discrepancies between these studies regarding the effects of RF could be, in part, linked to the time of day (TOD) of testing. However, this still remains open for investigation whether fasting or not alters the diurnal variation of physical performance of the fasting athletes.

On the other hand, limited attention has been paid to the cognitive impairments of athletes during RF (Ghayour Najafabadi et al., 2015; Chamari et al., 2016). Previous studies have demonstrated that reaction time (Roky et al., 2000; Bouhlel et al., 2014) and mental alertness (Boukhris et al., 2019a) were altered during RF. However, no adverse effects of fasting on specific cognitive performance were reported in athletes (Ghayour Najafabadi et al., 2015). In addition, cognitive function is also subject to the influence of circadian rhythm including the sleep–wake cycle (Ruiz-Gayo and Olmo, 2020; Khemila et al., 2021). In this sense, it has been observed that cognitive performance oscillates according to various temporal periodicities (Kline et al., 2010; Khemila et al., 2021). Interestingly, most studies examining the impact of fasting on cognitive function were conducted on rested athletes (Chamari et al., 2016). Ploughman (2008) indicated that cognitive performance could be modeled by the exercise intensity. However, whether high-intensity exercise would counteract the Ramadan-induced cognitive decline or not requires further research.

Specific recommendations on the best TOD to exercise/train during the month of Ramadan are contradictory. Further, the combined effect of RF and high intensity exercise on cognitive tasks at different TOD has not been studied. Therefore, it seems prudent to study the effects of RF on the diurnal variation of cognitive performances at rest and after exercise. It was hypothesized that during Ramadan (i) the diurnal variations in footballers’ performances would be flattened by a drop in these performances mainly in the afternoon and evening and not in the morning and (ii) that short-term maximal exercise may counteract the effect of fasting on cognitive function.

## Methods

### Participants

The software G*Power (Faul et al., 2007) was used to *a priori* calculate the least required sample size, based on procedures suggested by Beck (2013). Values for α were set at 0.05 and power at 0.80. Based on a study with similar paradigm (Graja et al., 2021) and discussions between the authors, effect sizes were estimated as 0.39. In total, to reach the desired power, data from at least 10 participants were deemed to be sufficient to minimize the risk of incurring a type 2 statistical error.

Eleven professional male football players (age: 19.27 ± 0.9 years.; height: 1.79 ± 0.04 m; body mass: 70.49 ± 3.97 kg; BMI: 21.81 ± 1.59 kg/m^2^) from the Tunisian league2 team volunteered to participate in this study. After receiving a description of the protocol, risks, and benefits of the study, each volunteer provided written informed consent prior to participation. Participants were active professional football players (8 ± 2 h of training/week) and maintained standard times for sleep according to their physiological sleep–wake cycle (sleeping between 22:30 h and 07:00 h ± 1:00 h) and for eating meals (breakfast at 07:00 h ± 1:00 h, lunch at 12:00 h ± 1:00 h, and dinner at 20:00 h ± 1:00 h) before Ramadan. None suffered from any injury or illness, all were non-smokers, non-habitual nappers, caffeine-naïve and medication-free. Participants began fasting at the age of 12.6 ± 0.7 years and were experienced with Ramadan observance (7.09 ± 0.70 years). Since circadian typology might affect the study outcomes, the selection of the participant’s chronotypes was based on the self-assessment questionnaires of Horne and Ösberg (1976) and only “neither type” players were recruited. Since the COVID-19 pandemic might affect mental health, physical activity, and quality of life of athletes (Romdhani et al., 2022a, b), only participants who have never been affected by the COVID-19 have been selected. The protocol of this study complied with Helsinki’s declaration for human experimentation and was approved by the local University Ethics Committee (CPP: N° 0117/2020).”

### Experimental design

**Figure 1:**
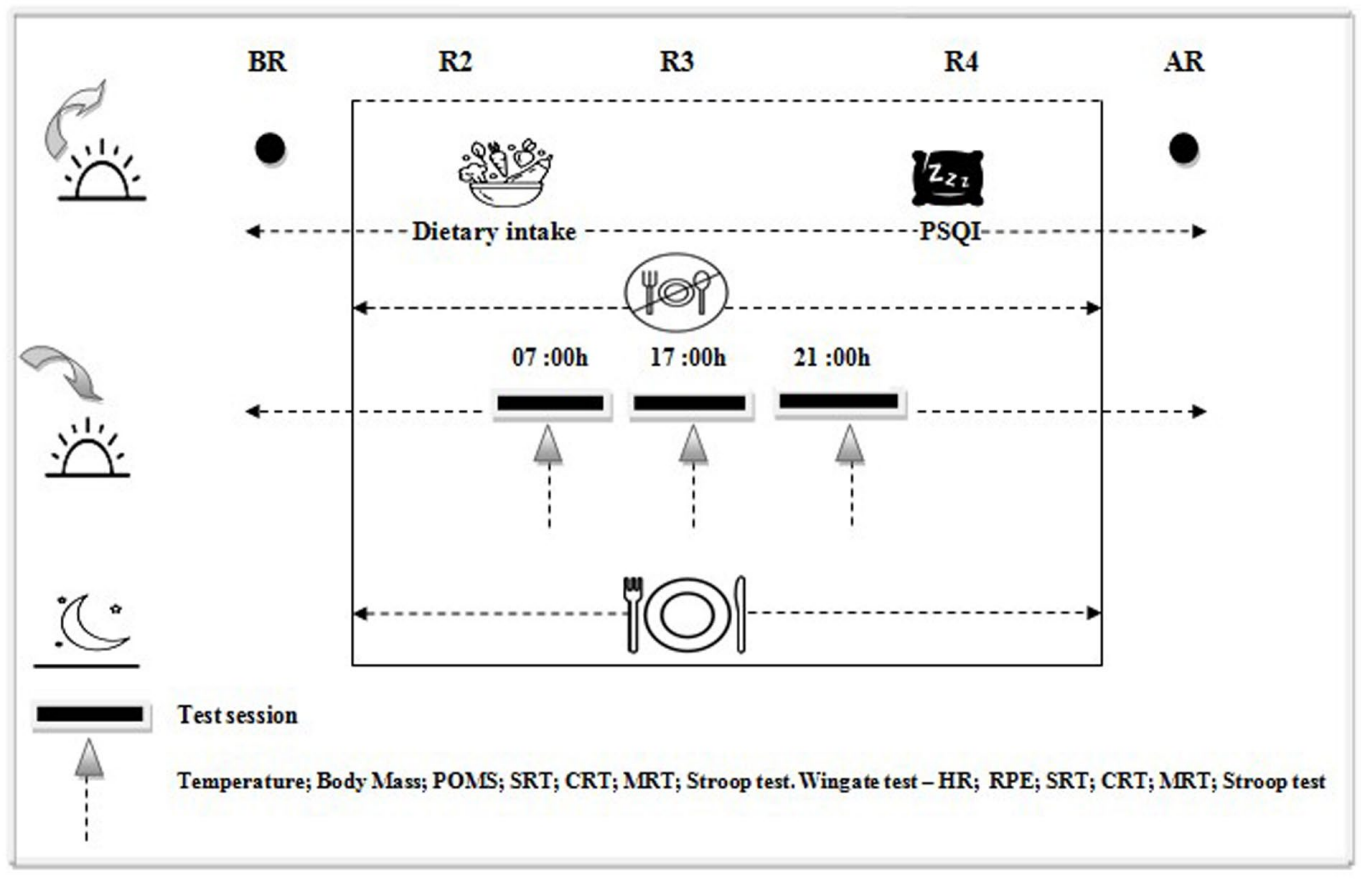
Study design. BR; 1 week before Ramadan, R2; the 2nd week of Ramadan, R3; the 3rd week of Ramadan, R4; the 4th week of Ramadan, AR; 2 weeks after Ramadan, PSQI; Pittsburgh Sleep Quality Index, SRT; Simple reaction time, CRT; Choice reaction time, MRT; Mental rotation test, POMS; Profile of Mood States, all times given are expressed in local time (GMT + 1 h).

The study was conducted in the Spring during the month of Ramadan in 2022, which began on 02 April 2022 and ended on 01 May. The length of each fasting day was approximately 16–17 h. The experimental design consisted of five testing periods after a period of training for the tests: 1 week before Ramadan (BR); the second (R2); the third (R3); and the fourth (R4) week of Ramadan; and 2 weeks after Ramadan (AR); with the test sessions performed at 3 different times of day (07:00, 17:00, and 21:00 h). Test sessions were completed in a counterbalanced design with only one test session per day, allowing at least 48 h for washout. Each test session started with oral temperature and anthropometric measurements. After a 10 min seated rest, oral temperature was measured with a digital clinical thermometer (Omron, Paris, France; accuracy +0.05°C) inserted sublingually for at least 3 min. Body mass was measured using an electronic scale (Tanita, Tokyo, Japan). Profile of mood state (POMS) was completed by each participant during the 5 testing periods. Rate of perceived exertion (RPE) was determined using the Borg scale immediately after the Wingate test. Heart rate (HR) was recorded during the Wingate test, using a Polar heart rate monitor (Polar Electro Oy, T61-coded, Hungary). Sleep measurement was assessed by the Pittsburgh Sleep Quality Index (PSQI; Buysse et al., 1989). During each test session, the participants performed, in the same order, SRT, CRT, MRT, and selective attention, before and after the Wingate test. Throughout the experimental period, participants were requested to maintain their habitual physical activity and to avoid strenuous activities (Figure 1).

### Dietary intake analysis

The participants were required to record their food and nutrients intakes in a diary over a span of 3 days for each week of physical testing, which was completed with an interview by an experienced dietician. Dietary records were analyzed by the same dietician using the Bilnut program (Nutrisoft, Cerelles, France) and the food-composition tables of the National Institute of Statistics of Tunis (1978)^1^ using a computerized nutrition system, the NUTRISOFT—BILNUT (Version. 2.01, Paris, France).

### The profile of mood states

The profile of mood states (POMS) questionnaire developed by McNair (1971) was used to collect data about the mood of participants during each test session. It is a self-report questionnaire consisting of 65 items that aims to measure different emotional conditions (including five negative moods (tension-anxiety, depression, anger-hostility, vigor-activity, fatigue and confusion-bewilderment), one positive mood (vigor) and interpersonal relationships) and a total mood disturbances score (TMD) for which all scales, except interpersonal relationships, were taken into account [TMD = (Tension + Depression + Anger + Fatigue + Confusion) – Vigor]. Five-point Likert scales ranging from 0 (“not at all”) to 4 (“extreme”), in relation to the context of each item, were used.

### The Pittsburgh sleep quality index

The Pittsburgh sleep quality index (PSQI) was used to evaluate subjective sleep quality over the preceding month (Buysse et al., 1989). The PSQI includes 19 questions grouped into seven components: sleep quality, sleep duration, sleep latency, sleep efficiency, sleep disturbances, daytime dysfunction and the use of sleeping medications. The PSQI total score ranges from 0 to 21, with “0” indicating no troubles and “21” indicating severe problems in all areas of sleep. A PSQI score of ≥5 and ≥8 indicate poor and very poor sleep quality, respectively.

### Wingate test

The Wingate test was conducted on a friction-loaded cycle ergometer (Monark 894 E, Stockholm, Sweden) interfaced with a microcomputer, using optimization tables (0.087 kg kg^−1^ body mass; Bar-Or, 1987). Peak (PP) and mean (MP) powers were retained from the Wingate test and the Fatigue Index was calculated as follows:

Fatigue Index (%) = [(peak power − lowest power)/peak power] × 100.

### Rating of perceived exertion scale

Rating of perceived exertion (RPE) scale is a reliable indicator of physical discomfort, has sound psychometric properties and is strongly correlated with several other physiological measures of exertion. The score on the 10-point scale of Borg (1982) ranges from 0 (nothing at all; indicative of how you feel when sitting in a chair) to 10 (very, very heavy; indicative of how you feel at the end of a very, very difficult activity).

### Psychomotor and cognitive function

#### Simple reaction time and choice reaction time

As described by Khemila et al. (2021) participants performed simple and choice reaction time (CRT) using the Reaction, INRP free software (version 4.05) created by Tilquin.

#### Mental rotation test

Participants were asked to compare two samples of stimuli displayed on a computer screen and to decide whether they were identical. The stimuli used in this test were based on data provided by Shepard and Metzler (1971). The Open Sesame software (version 3.1) was used to conduct the test (Mathôt et al., 2012).

#### Selective attention

As determined by Khemila et al. (2021), there were three major boards to the Stroop test corresponding to three conditions (i.e., “word task,” “color task” and “color-word task”), each comprising a row of four lines and six rows of four colors stimuli. Performance was assessed based on both the time to complete each condition and the number of errors.

### Statistical analyses

Statistical analysis was performed using STATISTICA Software (StatSoft, France) and figures were created using GraphPad Prism 8 (GraphPad Software, San Diego, CA, United States). The Shapiro–Wilk test of normality revealed that the data were normally distributed; therefore, parametric tests were performed. PSQI and data of dietary intake (the average of the3 days) were analyzed using a one-way ANOVA [5 (testing periods)]. Data of (Oral temperature, body mass, BMI, POMS, Wingate test, HR and RPE) were analyzed using a two-way ANOVA [5 (Ramadan) × 3 (time-of-day)] with repeated measures. A three-way ANOVA for repeated measures was performed to determine the effects of time of day, Ramadan and exercise on the cognitive function (SRT, CRT, MRT and selective attention). To assess the ANOVA practical significance of data, effect sizes were calculated as partial eta-squared (𝜂_p_^2^). When appropriate, significant differences between means were tested using the Least Significant Different (LSD) Fisher’s post-hoc test. The effect size (d) was calculated according to Cohen (1992), to determine the magnitude of pairwise comparison. Statistical significance for all analyses was set at *p* ≤ 0.05. Data are presented as mean ± standard deviation (SD).

## Results

### Body composition and oral temperature

The ANOVA showed a significant effect of TOD [*F*_(1.11)_ = 16.9; *p* < 0.001; η_p_^2^ = 0.62] and Ramadan [F_(1.11)_ = 2.6; *p* < 0.05; η_p_^2^ = 0.20] on body temperature. Temperature values increased from the morning to the evening BR (*p* < 0.001; d = 1.43), R2 (*p* < 0.01; d = 1.47), R3 (*p* < 0.01; d = 1.19), R4 (*p* < 0.05; d = 0.87), and AR (*p* < 0.01; d = 1.91). There was a significant decrease in body temperature at R3 (*p* < 0.05; d = 0.75) and R4 (*p* < 0.01; d = 0.86) in the afternoon in comparison with BR. However, no significant effect of Ramadan or TOD (*p* > 0.05) on body mass and body mass index (BMI) was detected (Table 1).

**Table 1:** Values (mean ± SD) of body mass, body mass index (BMI), and oral temperature recorded during the three times of the day (07:00, 17:00, and 21:00 h) 1 week before Ramadan (BR), during the 2nd week of Ramadan (R2), in the 3rd week of Ramadan (R3), during the 4th week of Ramadan (R4) and 2 weeks after Ramadan (AR; *n* = 11).

		Body mass (kg)	Body mass index (Kg/m^2^)	Oral temperature
BR	07:00 h	72.58 ± 6.90	22.43 ± 2.12	36.04 ± 0.63
	17:00 h	72.52 ± 6.89	22.42 ± 2.11	36.79 ± 0.72^***^
	21:00 h	72.59 ± 6.90	22.44 ± 2.13	36.80 ± 0.40^***^
R2	07:00 h	72.76 ± 7.10	22.49 ± 2.14	36.20 ± 0.33
	17:00 h	72.67 ± 6.84	22.46 ± 2.10	36.60 ± 0.27^*^
	21:00 h	72.76 ± 6.72	22.49 ± 2.09	36.70 ± 0.34^**^
R3	07:00 h	72.75 ± 6.73	22.49 ± 2.05	36.13 ± 0.41
	17:00 h	72.70 ± 6.90	22.47 ± 2.15	36.37 ± 0.29^#^
	21:00 h	72.75 ± 6.91	22.49 ± 2.12	36.61 ± 0.38^**^
R4	07:00 h	72.73 ± 6.56	22.48 ± 2.04	36.10 ± 0.52
	17:00 h	72.70 ± 6.67	22.47 ± 2.07	36.30 ± 0.33^##^
	21:00 h	72.73 ± 6.89	22.49 ± 2.11	36.50 ± 0.38^*^
AR	07:00 h	72.67 ± 6.90	22.46 ± 2.11	36.00 ± 0.23
	17:00 h	72.71 ± 6.81	22.47 ± 2.08	36.60 ± 0.17 ***
	21:00 h	72.71 ± 6.80	22.47 ± 2.08	36.50 ± 0.28 **

BR, 1 week before Ramadan; R2, 2nd week of Ramadan; R3, 3rd week of Ramadan; R4, 4th week of Ramadan; AR, 2 weeks after Ramadan. *, **, ***: significant difference in comparison with the morning at *p* < 0.05, *p* < 0.01, *p* < 0.001, respectively; #, ##, ###: significant difference in comparison with BR in the afternoon at *p* < 0.05, *p* < 0.01, *p* < 0.001, respectively.

### Dietary intake

Comparison of daily mean energy and macro-nutrient intakes by the participants in the 5 different weeks showed no significant statistical differences (*p* > 0.05; Table 2).

**Table 2:** Mean values ± standard deviation (SD) of estimated daily dietary intake recorded 1 week before Ramadan (BR), in the 2nd week of Ramadan (R2), in the 3rd week of Ramadan (R3), in the 4th week of Ramadan (R4), and 2 weeks after Ramadan (AR; *n* = 11).

	BR	R2	R3	R4	AR
Energy intake (kcal/day)	1922.84 ± 214.30	1912.72 ± 291.33	1978.72 ± 374.80	2117.72 ± 453.33	2091.72 ± 266.07
Carbohydrate (%)	215.27 ± 58.69	223.00 ± 49.34	235.54 ± 49.34	237.27 ± 63.08	225.36 ± 77.08
Fat (%)	71.69 ± 29.72	54.90 ± 16.47	57.81 ± 22.29	60.18 ± 18.20	71.27 ± 25.34
Protein (%)	70.32 ± 13.27	70.45 ± 21.45	74.81 ± 20.13	74.63 ± 25.12	75.90 ± 14.80

### Profile of mood states

A significant interaction Ramadan × TOD was observed for fatigue [*F*_(8.80)_ = 2.26; *p* < 0.05; η_p_^2^ = 0.18], anxiety [F_(8.80)_ = 2.12; *p* < 0.05; η_p_^2^ = 0.17] and vigor [F_(8.80)_ = 3.44; *p* < 0.01; η_p_^2^ = 0.25]. Anxiety scores (*p* < 0.001; d = 1.82; d = 1.72, respectively) and fatigue scores (*p* < 0.05, d = 1.36; *p* < 0.001, d = 0.85, respectively) were higher and vigor scores (*p* < 0.01, d = 1.82; d = 2.35, respectively) were lower during R3 and R4 in comparison with BR only in the afternoon (Table 3).

**Table 3:** Values (mean ± SD) of anxiety, anger, confusion, depression, fatigue, vigor, interpersonal relationship and total mood disturbances (TMD) scores registered by the Profile of Mood State (POMS) questionnaire during the three times of the day (07:00, 17:00, and 21:00 h) 1 week before Ramadan (BR), during the 2nd week of Ramadan (R2), the 3rd week (R3), the 4th week of Ramadan (R4) and 2 weeks after Ramadan (AR; *n* = 11).

		Anxiety	Anger	Confusion	Depression	Fatigue	Vigor	Interpersonal relationship	Total mood disturbances (TMD)
BR	07:00 h	3.09 ± 1.22	5.90 ± 3.85	4.72 ± 2.05	3.90 ± 3.17	2.89 ± 2.62	18.18 ± 3.09	18.20 ± 6.91	5.70 ± 9.06
	17:00 h	3.09 ± 0.94^***###^	7.45 ± 4.20	4.36 ± 4.00	5.09 ± 7.54	2.72 ± 2.14^*###^	19.00 ± 2.93^**##^	18.80 ± 6.82	3.72 ± 15.90^#+^
	21:00 h	3.63 ± 2.01	4.00 ± 2.60	4.27 ± 4.51	3.18 ± 4.11	3.27 ± 2.83	21.18 ± 5.26	16.81 ± 10.87	−2.18 ± 10.45
R2	07:00 h	3.72 ± 0.64	4.00 ± 3.84	4.63 ± 2.24	2.45 ± 2.16	2.54 ± 1.80	19.00 ± 5.93	13.09 ± 8.43	−1.63 ± 7.61
	17:00 h	3.63 ± 1.36^**##^	4.00 ± 2.32	3.54 ± 2.06	2.36 ± 2.90	2.63 ± 1.28^*###^	18.18 ± 8.51^**##^	16.45 ± 7.67	−2.00 ± 11.43^**###^
	21:00 h	3.72 ± 1.61	4.09 ± 1.75	3.81 ± 1.40	2.54 ± 3.20	2.36 ± 2.87	20.72 ± 3.97	19.18 ± 5.92	−4.18 ± 7.93
R3	07:00 h	3.63 ± 1.50^**^	3.90 ± 3.41	3.09 ± 1.75	2.63 ± 1.91	2.90 ± 2.84^*^	18.63 ± 6.08^**^	14.72 ± 7.92	−2.45 ± 8.84^**^
	17:00 h	6.27 ± 2.2	3.54 ± 3.41	3.18 ± 2.78	2.18 ± 1.77	5.54 ± 1.96	12.72 ± 3.87	12.36 ± 7.65	8.00 ± 6.82
	21:00 h	3.90 ± 2.07^**^	3.36 ± 2.61	3.09 ± 2.34	3.27 ± 3.00	2.54 ± 2.38^**^	19.18 ± 6.01^**^	18.18 ± 9.90	−2.00 ± 9.05^**^
R4	07:00 h	3.45 ± 4.22^##^	6.54 ± 4.15	3.90 ± 1.13	3.63 ± 4.10	2.54 ± 2.33^###^	18.00 ± 6.09^##^	15.18 ± 6.22	2.09 ± 16.90^##^
	17:00 h	6.18 ± 2.35	5.45 ± 3.95	4.09 ± 2.98	2.45 ± 2.58	6.18 ± 6.40	12.45 ± 2.62	14.72 ± 7.63	12.54 ± 15.35
	21:00 h	3.45 ± 2.38^##^	4.36 ± 3.93	3.27 ± 1.42	3.27 ± 3.40	2.72 ± 3.60^###^	18.18 ± 6.33^##^	16.54 ± 9.45	−1.09 ± 14.76^###^
AR	07:00 h	3.09 ± 1.37	3.72 ± 2.57	2.27 ± 1.55	2.63 ± 2.50	2.63 ± 3.61	17.09 ± 4.54+	18.45 ± 6.26	−2.72 ± 9.02
	17:00 h	3.81 ± 1.99^**##^	4.00 ± 2.04	3.18 ± 1.77	2.09 ± 1.64	2.54 ± 1.80^**###^	21.81 ± 5.03^***###^	19.63 ± 4.96	−6.18 ± 7.89^***###^
	21:00 h	3.36 ± 1.62	4.18 ± 6.44	3.36 ± 3.23	2.72 ± 2.76	2.63 ± 2.20	13.36 ± 7.18+	13.36 ± 7.31	−1.09 ± 15.94

BR, Before Ramadan; R2, second week of Ramadan; R3, third week of Ramadan; R4, fourth week of Ramadan; AR, After Ramadan.*, **, ***: significant difference in comparison withR3 in the afternoon at *p* < 0.05, *p* < 0.01, *p* < 0.001, respectively; #, ##, ###: significant difference in comparison with R4 in the afternoon at *p* < 0.05, *p* < 0.01, *p* < 0.001, respectively;. +, ++, +++: significant difference in comparison with AR in the afternoon at *p* < 0.05, *p* < 0.01, *p* < 0.001, respectively.

### The Pittsburgh sleep quality index

ANOVA revealed a significant effect of Ramadan on subjective sleep quality [*F*_(4.45)_ = 8.75; *p* < 0.001; η_p_^2^ = 0.43], sleep duration [*F*_(4.45)_ = 10.44; *p* < 0.001; η_p_^2^ = 0.48], and sleep latency [*F*_(4.45)_ = 12.88; *p* < 0.001; η_p_^2^ = 0.53]. Sleep quality was lower BR in comparison to R2 (*p* < 0.01; d = 1.60), R3 (*p* < 0.001; d = 1.93), R4 (*p* < 0.001; d = 2.03) and AR (*p* < 0.01; d = 1.44). Sleep duration was higher during R3 and R4 compared to BR (*p* < 0,001, d = 1.74; d = 2.19, respectively) and during R4 than R2 and AR (*p* < 0.05, d = 1.12; *p* < 0.01, d = 1.61, respectively). Sleep latency was shorter during BR relative to R3 and R4 (*p* < 0.001; d = 1.29; d = 1.78, respectively) and AR compared to R3 and R4 (*p* < 0.01, d = 0.91; *p* < 0.001, d = 1.29, respectively; Table 4).

**Table 4:** PSQI questionnaire parameters (mean ± SD) recorded 1 week before Ramadan (BR), at the 2nd week of Ramadan (R2), at the 3rd week of Ramadan (R3), during the 4th week of Ramadan (R4) and 2 weeks after Ramadan (AR; *n* = 11).

	BR	R2	R3	R4	AR
Subjective sleep quality	0.80 ± 0.63	1.80 ± 0.63^**^	2.00 ± 0.47^***^	2.20 ± 0.63^***^	1.70 ± 0.48^**^
Sleep latency (au)	0.60 ± 0.51	1.30 ± 0.48^¤¤^	2.20 ± 0.63^***++^	2.50 ± 0.52^***^	1.00 ± 1.15^¤¤¤^
Sleep duration (au)	0.60 ± 0.69	1.30 ± 0.67^¤^	1.80 ± 0.42^***^	2.20 ± 0.78^***^	1.10 ± 0.31^¤¤^
Sleep efficiency%	97% ± 0.05	95% ± 0.06	94% ± 0.09	93% ± 0.11	96% ± 0.06
Sleep disturbance	0.50 ± 0.52	0.60 ± 0.69	0.80 ± 0.63	0.90 ± 0.56	0.70 ± 0.48
Use of sleeping medication	0.00 ± 0.00	0.00 ± 0.00	0.00 ± 0.00	0.00 ± 0.00	0.00 ± 0.00
Day time dysfunction	0.40 ± 0.51	0.60 ± 0.69	0.80 ± 0.78	0.90 ± 0.73	0.50 ± 0.52
Global score PSQI	3.78 ± 0.30	6.55 ± 0.59	8.54 ± 0.79	9.63 ± 0.92	5.96 ± 0.53

AU, arbitrary unit; BR, Before Ramadan; R2, second week of Ramadan; R3, third week of Ramadan; R4, fourth week of Ramadan; AR, After Ramadan. *, **, ***: significant difference in comparison with BR at *p* < 0.05, *p* < 0.01, *p* < 0.001, respectively; +, ++, +++: significant difference in comparison with AR at *p* < 0.05, *p* < 0.01, *p* < 0.001, respectively; ¤, ¤¤, ¤¤¤: significant difference in comparison with R4 at *p* < 0.05, *p* < 0.01, *p* < 0.001, respectively.

### Wingate test mean and peak powers

#### Mean power

There was a significant effect of TOD [*F*_(2.20)_ = 6.64; *p* < 0.01; ηp2 = 0.39] and Ramadan [*F*_(4.40)_ = 10.05; *p* < 0.001; η_p_^2^ = 0.50] on mean power (MP). MP was higher in the afternoon than in the morning during BR, R2 and AR (*p* < 0.01, d = 0.94; d = 1.36; d = 1.21, respectively) and was higher in the evening than in the morning during BR, R2 and AR (*p* < 0.01, d = 0.88; d = 1.53; d = 1.22, respectively). Moreover, MP decreased during R3 and R4 at17:00 h in comparison with BR (*p* < 0.05; d = 1.18; *p* < 0.001, d = 2.21, respectively) and with AR (*p* < 0.05; d = 1.03; *p* < 0.001, d = 2.06, respectively). MP decreased during R4 in comparison to BR and AR at 21:00 h (*p* < 0.01; d = 0.95; respectively). However, no significant differences were observed in MP during Ramadan at 07:00 h (*p* > 0.05).

#### Peak power

There was a significant effect of TOD [*F*_(2.20)_ = 26.31; *p* < 0.001; η_p_^2^ = 0.72] and of Ramadan [*F*_(4.40)_ = 9.73; *p* < 0.001; η_p_^2^ = 0.49] on peak power (PP). PP was higher in the afternoon compared to the morning during BR, R2, R3 and AR (*p* < 0.01, d = 1.35; d = 0.71; d = 1.10; d = 1.28, respectively) and was higher in the evening than in the morning during BR, R2, R3 and AR (*p* < 0.001; d = 1.44; *p* < 0.01, d = 1.15; *p* < 0.05, d = 1.24; *p* < 0.01, d = 1.72, respectively). In comparison with BR, PP decreased during R3 and R4 at 17:00 h (*p* < 0.05; d = 1.14; *p* < 0.001, d = 1.77, respectively) and 21:00 h (*p* < 0.01; d = 1.30; *p* < 0.001, d = 2.05, respectively). Moreover, PP increased during AR in comparison with R4 in the afternoon and in the evening (*p* < 0.001; d = 1.50; d = 2.14, respectively). However, no significant differences were observed in PP during Ramadan at 07:00 h.

#### Heart rate

There was a significant Ramadan × TOD interaction [*F*_(8.80)_ = 3.12; *p* < 0.01; η_p_^2^ = 0.23] on heart rate (HR). HR were higher in the afternoon and evening compared to the morning BR (*p* < 0.05, d = 0.25; d = 0.24, respectively). In comparison with BR, HR decreased during R2, R3 and R4 in the afternoon (*p* < 0.01; d = 0.50; d = 0.56; d = 0.84, respectively) and evening (*p* < 0.001, d = 0.051; d = 0.58; d = 0.85, respectively). In addition, HR were lower during R2, R3 and R4 compared to AR in the afternoon (*p* < 0.001, d = 0.45; d = 0.51, d = 0.80, respectively) and evening (*p* < 0.001; d = 0.39, d = 0.45, d = 0.73, respectively).

#### Rating of perceived exertion scale

There was a significant effect for Ramadan [*F*_(4.40)_ = 3.24; *p* < 0.05; η_p_^2^ = 0.24] and for TOD [*F*_(2.20)_ = 10.11; *p* < 0.001; η_p_^2^ = 0.50]. Rating of perceived exertion (RPE) scores were higher in the afternoon than in the morning during R2, R3 and R4 (*p* < 0.01; d = 0.95; d = 1.12; d = 1.58, respectively) and in the evening (*p* < 0.01, d = 0.99; d = 0.93; d = 1.58, respectively). RPE scores were lower BR than in the R2, R3 and R4 (*p* < 0.01; d = 0.96; d = 1.12; d = 1.89, respectively) only in the afternoon. Moreover, RPE scores were higher in R2 (*p* < 0.05, d = 0.80), R3 (*p* < 0.05, d = 0.93) and R4 (*p* < 0.01, d = 1.57) compared with AR only in the afternoon (Table 5).

**Table 5:** Values (mean ± SD) of Wingate scores [Peak power (PP), Mean power (MP), and fatigue index (FI)], heart rate (HR) and rating of perceived exertion scores (RPE) registered during the three times of the day (07:00, 17:00, and 21:00 h) before Ramadan (BR), the second week of Ramadan (R2), the third week of Ramadan (R3), the fourth week of Ramadan (R4) and after Ramadan (AR; *n* = 11).

		PP (Watts)	MP (Watts)	FI (Watts.S^−1^)	RPE (au)	HR (bpm)
BR	07:00 h	10.91 ± 0.23	8.12 ± 0.42	46.44 ± 2.79	6.90 ± 0.31	190.94 ± 1.69
	17:00 h	11.92 ± 0.22^**^	9.15 ± 0.20^**^	48.95 ± 1.89	7.18 ± 0.26	192.36 ± 1.68^*^
	21:00 h	12.01 ± 0.23^***^	9.07 ± 0.17^**^	48.84 ± 2.18	7.09 ± 0.39	192.26 ± 1.58^*^
R2	07:00 h	10.61 ± 0.34	8.02 ± 0.20	49.06 ± 1.78	7.09 ± 0.34^¤¤^	190.84 ± 1.56^…^
	17:00 h	11.54 ± 0.44^**…^	8.96 ± 0.21^**…^	53.22 ± 1.41	8.36 ± 0.45^##+^	189.64 ± 1.54^*##.+++^
	21:00 h	11.57 ± 0.11^**.^	9.04 ± 0.20^**.^	48.78 ± 2.20	7.18 ± 0.22^¤¤^	189.50 ± 1.63^*###.+++^
R3	07:00 h	10.37 ± 0.22	8.01 ± 0.21	51.81 ± 1.99	7.18 ± 0.26^¤¤^	189.90 ± 1.54
	17:00 h	11.13 ± 0.20^*#.^	8.37 ± 0.20^#.+^	54.28 ± 1.58	8.36 ± 0.36^##+^	189.44 ± 1.40#^##.+++^
	21:00 h	11.16 ± 0.16^*##+^	8.61 ± 0.22	49.14 ± 1.91	7.36 ± 0.27^¤^	189.33 ± 1.43^###.+++^
R4	07:00 h	10.34 ± 0.15	7.91 ± 0.33	48.21 ± 1.52	7.27 ± 0.30^¤¤^	188.95 ± 1.53^###^
	17:00 h	10.33 ± 0.31^###^	7.61 ± 0.22^###^	58.11 ± 3.87^**##^	8.54 ± 0.15^##++^	187.96 ± 1.46^###^
	21:00 h	10.70 ± 0.15^###^	8.09 ± 0.40^##^	54.34 ± 2.32	7.45 ± 0.24^¤^	187.93 ± 1.45^###^
AR	07:00 h	10.82 ± 0.19	8.10 ± 0.27	48.64 ± 1.27	7.09 ± 0.36	190.85 ± 1.66^…^
	17:00 h	11.68 ± 0.22^**..^	9.06 ± 0.20^**…^	52.73 ± 4.46^**^	7.36 ± 0.27	191.92 ± 1.49^…^
	21:00 h	11.83 ± 0.17^**…^	9.14 ± 0.24^**.^	48.89 ± 1.32^**^	7.18 ± 0.22	191.60 ± 1.554^…^

BR, Before Ramadan; R2, second week of Ramadan; R3, third week of Ramadan; R4, fourth week of Ramadan; AR, After Ramadan. *, **, ***: Significant difference from morning at *p* < 0.05, *p* < 0.01, *p* < 0.001, respectively ¤, ¤¤, ¤¤¤: significant difference from afternoon at *p* < 0.05, *p* < 0.01, *p* < 0.001, respectively. #, ##, ###: Significant difference from BR at same time of day at *p* < 0.05, *p* < 0.01, *p* < 0.001, respectively; #, ##, ###: Significant difference from R4 at same time of day at *p* < 0.05, *p* < 0.01, *p* < 0.001, respectively; +, ++, +++: Significant difference from AR at same time at *p* < 0.05, *p* < 0.01, *p* < 0.001.

### Psychomotor and cognitive function

#### Simple reaction time

There was a significant Ramadan × Exercise interaction [*F*_(4.40)_ = 3.98; p < 0.01; η_p_^2^ = 0.28]. SRT was higher in the morning compared to the afternoon and the evening before (*p* < 0.01, d = 0.77; d = 0.80, respectively) and after exercise (*p* < 0.01, d = 0.78; d = 0.83, respectively) BR and AR before exercise (*p* < 0.01, d = 0.89; d = 0.86) and after exercise (*p* < 0.01, d = 0.98; *p* < 0.05, d = 0.76).

Regarding the effect of Ramadan, Simple reaction time (SRT) increased during R2 and R3 in the afternoon before exercise compared to BR (*p* < 0.001, d = 1.04; d = 0.73, respectively) and AR (*p* < 0.001, d = 1.01; *p* < 0.01, d = 0.71, respectively). SRT increased during R2 and R3 in the evening before exercise compared to BR (*p* < 0.001, d = 1.15; d = 1.32, respectively) and AR (*p* < 0.01, d = 1.07; d = 1.26, respectively). However, exercise enhanced SRT in the afternoon and evening during R2 (*p* < 0.01, d = 0.98; d = 1.15, respectively) and R3 (*p* < 0.01, d = 0.73; d = 1.35, respectively).

#### Choice reaction time

There was only a significant effect of TOD [*F*_(2.20)_ = 24.09; *p* < 0.001; η_p_^2^ = 0.70] on CRT. Choice reaction time (CRT) was higher in the morning compared to the afternoon and evening before (*p* < 0.05, d = 0.87; d = 0.85, respectively) and after exercise (*p* < 0.05, d = 1.03; d = 1.10, respectively) BR and before exercise (*p* < 0.05, d = 1.43; d = 1.74) and after exercise (*p* < 0.05, d = 1.57; d = 0.81) AR.

#### Mental rotation test

##### The processing time of correct answers (MRT time)

There was only a significant effect of TOD [*F*_(2.20)_ = 8.51; *p* < 0.01; η_p_^2^ = 0.45] on Mental rotation test time (MRTT). MRTT was higher in the morning compared to the afternoon and evening before (*p* < 0.01, d = 1.68; d = 1.66, respectively) and after exercise (*p* < 0.05, d = 1.33; d = 1.30, respectively) BR and before exercise (*p* < 0.01, d = 1.81; d = 1.26) and after exercise (*p* < 0.05, d = 0.68; d = 0.56) AR.

##### Number of correct answers (MRTE)

There was a significant effect for TOD [*F*_(2.20)_ = 38.51; *p* < 0.001; η_p_^2^ = 0.79] and Ramadan × Exercise interaction [*F*_(4.40)_ = 5.54; *p* < 0.01; η_p_^2^ = 0.35]. MRTE was higher in the morning compared to the afternoon and evening before (*p* < 0.05, d = 0.94; d = 0.94, respectively) and after exercise (*p* < 0.05, d = 1.05; d = 0.93, respectively) BR, and before exercise (*p* < 0.05, d = 1.01; d = 1.13) and after exercise (*p* < 0.05, d = 1.30; d = 1.42) AR.

Regarding the effects of Ramadan, MRTE increased during R2 and R3 in the afternoon before exercise compared to BR (*p* < 0.05, d = 1.04; *p* < 0.01, d = 1.22, respectively) and AR (*p* < 0.01, d = 1.05; d = 1.20, respectively). MRTE increased during R2 and R3 in the evening before exercise compared to BR (*p* < 0.05, d = 1.27; d = 1.38, respectively) and AR (*p* < 0.01, d = 1.36; d = 1.46, respectively). However, exercise enhanced MRTE in the afternoon and evening during R2 (*p* < 0.001, d = 1.40; d = 2.01, respectively) and R3 (*p* < 0.001, d = 1.83; d = 2.75, respectively; Table 6).

**Table 6:** Values (mean ± SD) of simple reaction time (SRT) and choice reaction time (CRT) measured during the three times of the day (07:00, 17:00, and 21:00 h) 1 week before Ramadan (BR), the 2nd week of Ramadan (R2), the 3rd week of Ramadan (R3), the 4th week of Ramadan (R4), and 2 weeks after Ramadan (AR; *n* = 11).

		SRT (ms)		CRT (ms)		MRT time (s)		MRT error	
		Before exercise	After exercise	Before exercise	After exercise	Before exercise	After exercise	Before exercise	After exercise
BR	07: 00 h	272.55 ± 48.75	272.63 ± 47.08	402.82 ± 58.02	401.72 ± 48.28	1.90 ± 0.47	1.70 ± 0.49	4.55 ± 0.93	4.00 ± 1.09
	17: 00 h	239.70 ± 34.15^**^	238.30 ± 39.62^**^	358.90 ± 40.31^*^	359.50 ± 31.39^*^	1.06 ± 0.51^**^	1.03 ± 0.50^*^	3.73 ± 0.80^*^	3.09 ± 0.50^*^
	21: 00 h	239.40 ± 31.32^**^	238.70 ± 32.94^**^	360.80 ± 37.68^*^	356.64 ± 31.63^*^	1.10 ± 0.48^**^	1.12 ± 0.38^*^	3.73 ± 0.80^*^	3.09 ± 0.83^*^
R2	07: 00 h	277.80 ± 34.01	273.20 ± 48.25	399.27 ± 58.68	402.10 ± 31.44	1.95 ± 0.45	1.82 ± 0.81	4.73 ± 1.10	4.27 ± 0.90
	17: 00 h	280.60 ± 43.89^##¤¤^	242.50 ± 32.69^**++^	369.40 ± 45.23	370.82 ± 45.64	1.62 ± 0.62	1.45 ± 0.52	4.73 ± 1.10^#¤¤^	3.09 ± 1.22^**+++^
	21: 00 h	279.30 ± 37.34^###¤¤^	241.60 ± 27.06^++^	390.64 ± 30.11	372.18 ± 42.62	1.65 ± 0.44	1.56 ± 0.38	4.73 ± 0.78^#¤¤^	3.18 ± 0.75^**+++^
R3	07: 00 h	278.20 ± 38.44	271.82 ± 44.38	409.30 ± 27.52	403.36 ± 49.79	1.99 ± 1.77	1.85 ± 1.35	4.82 ± 1.08	4.27 ± 1.00
	17: 00 h	278.18 ± 65.35^##¤¤^	241.00 ± 29.85^**++^	380.45 ± 74.00	386.18 ± 29.36	1.62 ± 0.77	1.51 ± 0.53	4.82 ± 0.98^##¤¤^	3.00 ± 1.00^**+++^
	21: 00 h	281.09 ± 31.63^###¤¤^	240.64 ± 28.19^*+++^	383.09 ± 45.36	371.91 ± 37.33	1.47 ± 0.50	1.47 ± 0.66	4.73 ± 0.64^#¤¤^	3.09 ± 0.53^**+++^
R4	07: 00 h	276.50 ± 29.06	276.45 ± 32.30	408.60 ± 40.85	408.55 ± 44.59	2.04 ± 2.58	1.89 ± 0.67	4.27 ± 1.00	3.82 ± 0.60
	17: 00 h	259.20 ± 27.96	258.90 ± 27.54	375.60 ± 30.46	374.45 ± 50.22	1.72 ± 0.67	1.68 ± 0.50	4.18 ± 0.60	3.73 ± 0.78
	21: 00 h	263.50 ± 53.57	254.30 ± 40.98	371.27 ± 93.74	370.00 ± 47.48	1.76 ± 1.01	1.67 ± 1.06	4.00 ± 0.77	3.45 ± 0.93
AR	07: 00 h	276.64 ± 45.11	274.09 ± 46.52	403.45 ± 19.45	403.55 ± 25.60	1.94 ± 0.45	1.74 ± 0.86	4.55 ± 0.82	4.09 ± 0.70
	17: 00 h	240.90 ± 34.17^**^	237.18 ± 25.28^**^	350.36 ± 48.59^*^	352.55 ± 37.99^*^	1.18 ± 0.37^**^	1.17 ± 0.79^*^	3.55 ± 1.12^*^	3.09 ± 0.83^*^
	21: 00 h	245.20 ± 24.79^**^	245.36 ± 25.50^*^	356.00 ± 33.08^*^	351.00 ± 88.05^*^	1.16 ± 0.74^**^	1.17 ± 0.29^*^	3.55 ± 0.93^*^	3.09 ± 0.70^*^

BR, before Ramadan; R2, second week of Ramadan; R3, third week of Ramadan; R4, fourth week of Ramadan; AR, after Ramadan; SRT, simple reaction time; CRT, choice reaction time; MRT, mental test rotation; ms, milliseconds; s, second. *, **, *** significant difference in comparison with the morning at *p* < 0.05, *p* < 0.01, *p* < 0.001, respectively; +, ++, +++ significant effect of exercise at *p* < 0.05, *p* < 0.01, *p* < 0.001, respectively; #, ##, ### significant difference in comparison with BR at *p* < 0.05, *p* < 0.01, *p* < 0.001, respectively; ¤, ¤¤, ¤¤¤ significant difference in comparison with AR at *p* < 0.05, *p* < 0.01, *p* < 0.001, respectively.

##### Selective attention

There was a significant effect for TOD [*F*_(2.20)_ = 28.02; *p* < 0.001; η_p_^2^ = 0.73], Ramadan [*F*_(4.40)_ = 7.03; *p* < 0.001; η_p_^2^ = 0.41], and exercise [*F*_(1.10)_ = 22.36; *p* < 0.001; η_p_^2^ = 0.69]. Selective attention (SA) was higher in the morning compared to the afternoon and the evening before (*p* < 0.05, d = 1.00; d = 0.91, respectively) and after exercise (*p* < 0.01, d = 1.53; d = 1.27, respectively) BR, and before exercise (*p* < 0.01, d = 1.53; *p* < 0.05, d = 0.73) and after exercise (*p* < 0.01, d = 1.30; d = 1.23) AR.

**Figure 2:**
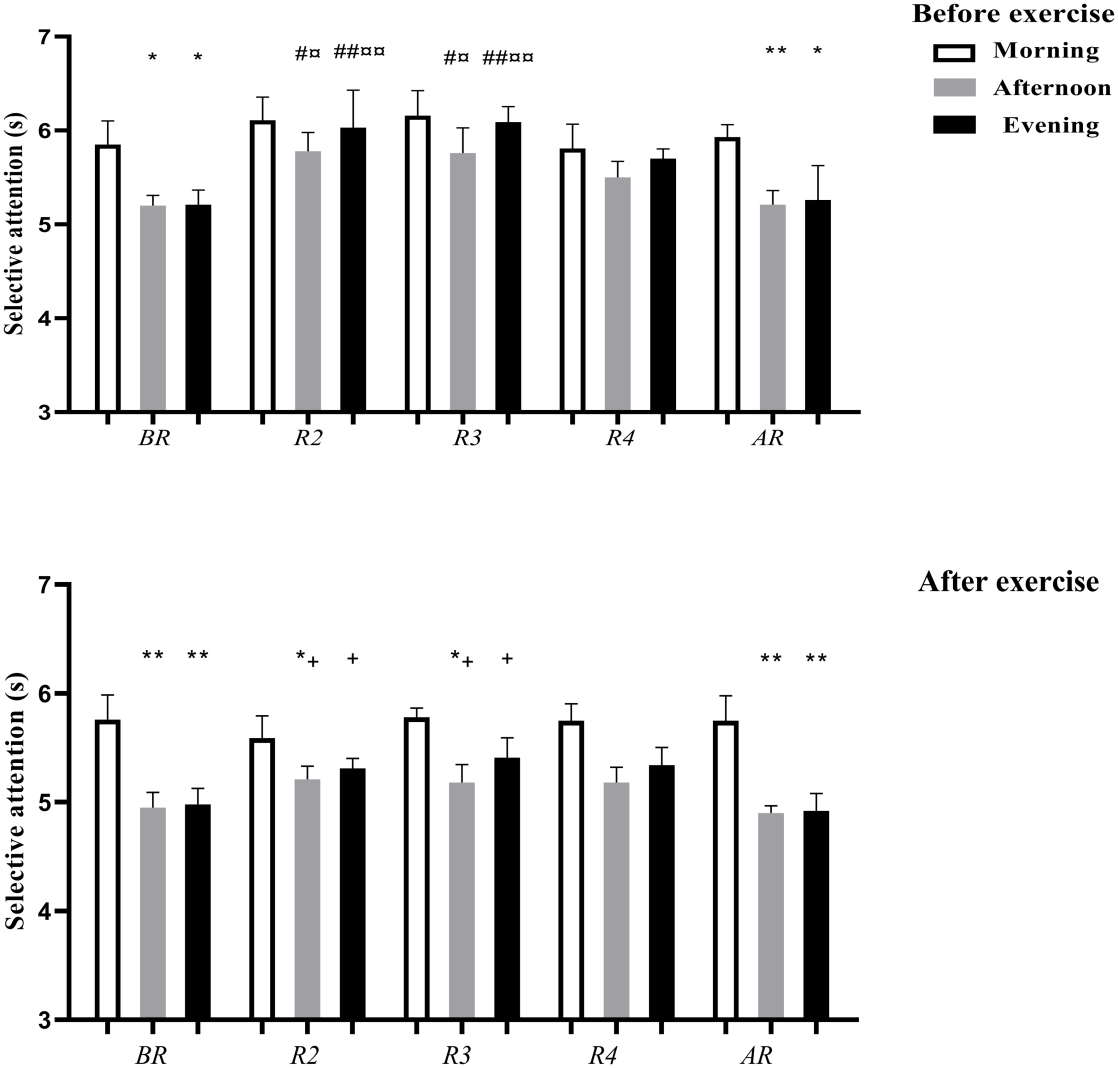
Selective attention (mean ± SD) measured before and after exercise in the morning, afternoon and evening 1 week before Ramadan (BR), the 2nd week of Ramadan (R2), the 3rd week of Ramadan (R3), the 4th week of Ramadan (R4) and 2 weeks after Ramadan (AR), *, **, ***: significant difference in comparison with the morning at *p* < 0.05, *p* < 0.01, *p* < 0.001, respectively; #, ##, ###: significant difference in comparison with before Ramadan at *p* < 0.05, *p* < 0.01, *p* < 0.001, respectively; ¤, ¤¤, ¤¤¤: significant difference in comparison with after Ramadan at *p* < 0.05, *p* < 0.01, *p* < 0.001, respectively; +, ++, +++: significant effects of exercise at *p* < 0.05, *p* < 0.01, *p* < 0.001, respectively.

Regarding the effects of Ramadan, SA increased during R2 and R3 in the afternoon before exercise compared to BR (*p* < 0.05, d = 1.09; d = 0.82, respectively) and AR (*p* < 0.05, d = 0.97; d = 0.76, respectively). SA increased during R2 and R3 in the evening before exercise compared to BR (*p* < 0.01, d = 1.32; d = 1.64, respectively) and AR (*p* < 0.01, d = 0.60; d = 0.87, respectively). However, exercise enhanced SA in the afternoon and in the evening during R2 (*p* < 0.05, d = 1.05; d = 0.68, respectively) and R3 (*p* < 0.05, d = 0.78; d = 1.18, respectively; Figure 2).

## Discussion

The major finding of this study was that RF altered the diurnal variation of short-term maximal performance and cognitive function of football players. During Ramadan, physical and cognitive performances were unaffected in the morning but were impaired in the afternoon and evening. Furthermore, short-term maximal exercise attenuates the negative impact of RF on cognitive performances.

### Effect of time of day

The present study found that peak (PP) and mean powers (MP) measured during the Wingate test were higher in the afternoon and evening compared to the morning before Ramadan (BR). These findings are in agreement with previous studies (Khemila et al., 2021; Bentouati et al., 2022), which reported that peak anaerobic performances were observed in the afternoon and evening. In addition, heart rate (HR) values were higher in the afternoon and evening compared to the morning. These results are consistent with previous research (Cruz-Martínez et al., 2015; Özçelik and Güvenç, 2016). Moreover, RPE scores are in agreement with those of Hammouda et al. (2013a) who did not show any diurnal variation of this parameter. Regarding cognitive performance, in agreement with previous studies (Jarraya and Jarraya, 2019; Souissi et al., 2019; Khemila et al., 2021), our results showed that SRT, CRT, MRT and selective attention were better in the evening and afternoon compared to the morning.

### Effect of Ramadan fasting

Regarding the effects of Ramadan, PP and MP decreased during the third (R3) and fourth week of Ramadan (R4) in the afternoon and evening but not in the morning compared to before Ramadan (BR) and after Ramadan (AR) and a significant increase in FI during R4 only in the afternoon compared to BR. These findings are in accordance with the results of previous data (Souissi et al., 2007; Chtourou et al., 2011). The lower performance in the evening during Ramadan could be due to the higher muscle fatigue at this TOD due to a lack of energy stores availability and the hydration status (Trabelsi et al., 2022a). Furthermore, some authors have suggested that the decrease in power during Ramadan may occur because participants are less motivated and less aroused (Souissi et al., 2007).

In fact, Ramadan is often accompanied by a sleep disturbance that could be the cause of the decline in performance (Romdhani et al., 2022c). In this context, our findings revealed that subjective sleep quality, sleep latency and sleep duration estimated by the PSQI scores were disrupted during RF compared to BR. These results confirm those of previous studies (Zerguini et al., 2007; Romdhani et al., 2022c) which found that subjective sleep quality was negatively affected during RF. It could be suggested that the disruption of sleep quality, sleep latency and sleep duration during Ramadan could be related to the delay in consumption of nocturnal meals and delay in nocturnal training or competitions which would similarly present a thermogenic effect on core body temperature and consequently promoted night wakefulness (Romdhani et al., 2022c). In contrast to these findings, others studies demonstrated no significant change in sleep duration in adolescent soccer players (Meckel et al., 2008) and in power athletes (Karli et al., 2007) as well as in the general population (Qasrawi et al., 2017). The heterogeneity concerning the effects of Ramadan on sleep in athletes could be related to differences in methodology, population, or to potential unidentified cultural differences.

In addition, our results showed that participants had higher fatigue and anxiety scores and lower vigor scores at R3 and R4 compared to BR in the POMS test. Similarly, previous studies have shown an increase in subjective feelings of fatigue, malaise, lethargy and mood alterations during Ramadan (Roky et al., 2004; Zerguini et al., 2007). These mood alterations could be explained by the lower sleep quantity and quality during Ramadan (Roky et al., 2004). Additionally, Roky et al. (2004) suggested mood disturbance and cognitive deterioration as the main reasons for reduced physical performance. Also, RPE scores were higher during Ramadan compared to BR and AR at 17:00 h. The increase in the perception of fatigue observed during Ramadan is consistent with a previous study (Chaouachi et al., 2009), potentially explained by these psychological factors. Hence, the drop in evening high-intensity performance could be related to the Ramadan-induced fatigue at this TOD.

In addition, HR during the Wingate test decreased during R2, R3 and R4 compared to BR and AR, in the afternoon and in the evening. These results are consistent with those of Hammouda et al. (2013b). It is well known that fasting is associated with catecholamine inhibition and reduced venous return, causing decreased sympathetic tone, which leads to decreased blood pressure, heart rate, and cardiac output (Suwaidi et al., 2006; Meckel et al., 2008). Thus, these physiological changes could be the cause of the decrease in performance during RF. Furthermore, RF has been reported to cause cumulative deficits in food and water intake (Trabelsi et al., 2018), but in the present study, daily energy intake did not change significantly during Ramadan compared to the pre-Ramadan control period. Our results confirm those of Aziz et al. (2011) and the findings of a recent systematic review (Boukhris et al., 2018).

For psychomotor and cognitive performances, we found that SRT, SA and number of correct answers (MRTE) increased in the afternoon and evening at rest during R2 and R3. These results are in agreement with previous reports (Doniger et al., 2006; Ghayour Najafabadi et al., 2015). Since glucose is a necessary substrate for the central nervous system and an increase in its metabolism has been demonstrated in particular areas of the brain during cognitive engagement (Chamari et al., 2016), the reduced performance that we found in the afternoon could be attributed to low blood glucose levels. In fact, the brain cannot produce its own glucose and must rely on constant peripheral input, which will be reduced in the late afternoon in fasting individuals. This drop in blood glucose in the afternoon during RF could be the source of the drop in cognitive performance.

However, RF had no effects on CRT and the processing time of correct answers (MRTT) at rest and after physical exercise in our participants. The lack of effect on these performances may be due to the different sensitivity of the various cognitive domains. In agreement with our results, Bouhlel et al. (2014) and Tian et al. (2011) reported that the effects of RF on cognitive performance were heterogeneous and domain-specific. These results may explain, at least in part, the unchanged cognitive performance (i.e., CRT and MRTT) in the present study.

Through our study, we also noticed that these cognitive performances tend to gradually recover from R4, testifying to the adaptation effect of the organism of footballers. This result confirms the finding of Lotfi et al. (2010) who proved that total reaction time and recognition reaction time were only altered at the beginning and not at the end of RF, suggesting a possible adaptation to intermittent fasting.

### Effect of exercise

Regarding the effect of physical exercise on the efficiency of psychomotor and cognitive processes, we observed that exercise intervened to counteract the fasting-induced increase (during R2 and R3) in SRT, MRTE and SA in the afternoon and evening. Consistent with our study, McMorris and Keen (1994) found a considerably slower SRT after performing exercise at 100% maximal aerobic power compared to exercising at 70% MAP in amateur athletes. Similarly, Lemmink and Visscher (2005) observed an improvement in CRT following intense intermittent exercise on an ergometer. These beneficial effects could be explained by the increase in catecholamine during maximal exercise which increases the space available for information processing, thus allowing greater efficiency (Sudo et al., 2017). Maximal exercise could therefore counteract the effect of RF on cognitive performances. The effects of physical exercise on cognitive performances during the month of Ramadan depend on the time of testing, diet, level of physical ability, cognitive task, and nature of the exercise (Lambourne and Tomporowski, 2010).

### Study limitations and implications for future research

The current study presents some limitations that need to be considered. The lack of non-fasting controls is typical of most studies of Ramadan, and reflects ethical issues in Muslim majority countries such as Tunisia. In such environments, it is unfortunately not possible to recruit non-fasting study participants. Therefore, in such cases, RF measurements are frequently compared to the pre-Ramadan baseline as reference values, as usually reported in the literature. Furthermore, sleep measurements were recorded only subjectively using the PSQI. Highlighting potential circadian disruptions through actigraphy or polysomnography is a more powerful tool to monitor sleep behavior. This may be an important aspect for future investigation. Finally, the lack of data on blood glucose measurements can be considered as another limitation of the present investigation. Consequently, further studies should replicate the study while controlling blood glucose. More physiological variables, such as blood pressure, lactate levels, and hormones (e.g., cortisol, adrenalin, and noradrenalin), must also be measured to validate the relationship between cognitive performance and the activation induced by physical exercise.

## Conclusion

Our results suggest that Ramadan fasting has no effects on morning’s physical and cognitive performances. However, physical and cognitive performances recorded in the afternoon and evening deteriorated significantly during Ramadan. Indeed, fluctuations in the performance of footballers were affected during Ramadan. It seems that the effect of time of day on physical and cognitive performance tends to disappear during Ramadan by the deterioration of performance recorded in the afternoon and evening. In addition, short-term maximal exercise mitigated the negative effects of fasting on cognitive performance.

## Data availability statement

The original contributions presented in the study are included in the article/supplementary material, further inquiries can be directed to the corresponding author.

## Ethics statement

The studies involving human participants were reviewed and approved by University of Manouba Ethics Committee. The patients/participants provided their written informed consent to participate in this study.

## Author contributions

SK and NS conceived the idea of conducting the present study, determined the study design, and executed the experimental sessions. SK computed and analyzed the data and prepared the first draft of manuscript. SK, MF, EB, and RA supervised the project. MR, SA, MF, MS, SG, and NS proofread the manuscript. MR, SG, and NS extensively examined the depth of the protocol, results, and discussion. All authors contributed to the article and approved the submitted version.

## Conflict of interest

The authors declare that the research was conducted in the absence of any commercial or financial relationships that could be construed as a potential conflict of interest.

## Publisher’s note

All claims expressed in this article are solely those of the authors and do not necessarily represent those of their affiliated organizations, or those of the publisher, the editors and the reviewers. Any product that may be evaluated in this article, or claim that may be made by its manufacturer, is not guaranteed or endorsed by the publisher.

## Footnotes

^1^ https://data.bnf.fr/fr/11864527/institut_national_de_la_statistique_tunisie/?fbclid=IwAR14-99nieeV572ADx3ZXqxsK46vY4KL9hPnJZQ8cBV3HPJPFt6NgzJlw3U
